# Including a pen and/or cover letter, containing social incentive text, had no effect on questionnaire response rate: a factorial randomised controlled Study within a Trial

**DOI:** 10.12688/f1000research.23767.2

**Published:** 2021-11-08

**Authors:** Sophie James, Adwoa Parker, Sarah Cockayne, Sara Rodgers, Caroline Fairhurst, David J. Torgerson, Sarah Rhodes, Sarah Cotterill

**Affiliations:** 1 York Trials Unit, University of York, UK, York, Y010 5DD, UK; 2 Centre for Biostatistics, University of Manchester, Manchester, M13 9PL, UK

**Keywords:** Retention, pen, social incentive, cover letter, randomised controlled trial, embedded trial, SWAT, postal questionnaire, response rate

## Abstract

**Background: **Postal questionnaires are frequently used in randomised controlled trials to collect outcome data on participants; however, poor response can introduce bias, affect generalisability and validity, and reduce statistical power. The objective of this study was to assess whether a pen and/or social incentive text cover letter sent with a postal follow-up questionnaire increased response rates in a trial.

**Method:** A two-by-two factorial randomised controlled trial was embedded within the OTIS host trial. Participants due their 12-month (final) follow-up questionnaire were randomised to be sent: a pen; a social incentive text cover letter; both; or neither. The primary outcome measure was the proportion of participants in each group who returned the questionnaire. Secondary outcomes were: time to return, completeness of the questionnaire, necessity of a reminder letter, and the cost effectiveness.

**Results: **The overall 12-month questionnaire response rate was 721 out of 755 (95.5%). Neither the pen nor social incentive cover letter had a statistically significant effect on response rate: pen 95.2% vs. no pen 95.8%, adjusted OR 0.90 (95% CI 0.45 to 1.80; p=0.77); social incentive cover letter 95.2% vs. no social incentive cover letter 95.8%, adjusted OR 0.84 (95% CI 0.42 to 1.69, p=0.63). No statistically significant differences were observed between either of the intervention groups on time to response, need for a reminder or completeness. Therefore, neither intervention was cost-effective.

**Conclusions: **We found no evidence of a difference in response rates associated with the inclusion of a pen and/or social incentive cover letter with the final follow-up postal questionnaire of the host trial. However, when these results are combined with previous SWATs, the meta-analysis evidence remains that including a pen increases response rates. The social incentive cover letter warrants further investigation to determine effectiveness.

**Trial registration: ISRCTN22202133 **(21st June 2020).

## Introduction

Randomised controlled trials (RCTs) are the gold standard to assess effectiveness of treatment options and to inform care decisions ^1^, yet only a few hundred studies exist to assess the effectiveness of different methods to improve retention or recruitment into RCTs ^2^.

Trial methodologists and funders have highlighted the need to evaluate participant recruitment and retention strategies in order to provide evidence on which to base decisions around the design and conduct of RCTs ^3^.

Postal questionnaires are frequently used in randomised controlled trials to collect outcome data on participants; however, poor response can introduce bias, affect generalisability and validity, and reduce statistical power. Several systematic reviews report on the topic of retention strategies, including improving response rates to questionnaires ^4– 7^. However, there remains a lack of definitive evidence regarding some commonly adopted practices such as sending a pen or using a cover letter with a questionnaire to encourage the participant to return it ^8– 10^. The results of a study within a trial (SWAT) evaluating these two strategies are reported here.

## Methods

### Design

A two-by-two factorial RCT was embedded within the OTIS trial of occupational therapist-led home assessment and modification for the prevention of falls ( ISRCTN22202133) ^11^. OTIS recruited participants over the age of 65 years who were at risk of falling. Participants were randomised to receive an occupational therapist delivered visit or usual care. They were followed up for 12 months for falls data and were sent postal questionnaires at four, eight and 12 months. This SWAT was embedded at the 12-month time point. Ethical approval for this SWAT was received from the NHS West of Scotland Research Ethics Committee 3 (16/WS/0154) and Health Research Authority and Research Ethics approval in July 2018. Approvals were obtained from the University of York, Department of Health Sciences Research Governance Committee. Participants provided informed consent to be enrolled into the OTIS trial and to be sent study related information by post. Consent for the SWAT was therefore waived by the above-named ethics committee. 

### Participants

A total of 779 participants due to receive their 12-month questionnaire between 16 ^th^ October 2018 and 2 ^nd^ August 2019 were randomised into the SWAT in a single tranche in September 2018. Participants who had withdrawn from the OTIS study prior to this were excluded from randomisation.

The allocation sequence was generated by the OTIS statistician, who was not involved with the sending of the questionnaires, using STATA v15 ^12^. The identification numbers of OTIS participants to be involved in the SWAT were randomised 1:1:1:1 in a single block. Because there were no descriptive details of the participants attached to the identification numbers this meant the randomisation was concealed.

### Interventions

Table 1 details the combination of interventions sent in the post with the 12-month questionnaire. We included an unconditional £5 note with the questionnaire for all participants.

The non-standard cover letter offered a mild level of social incentive, in the form of a personalised table that indicated whether or not a questionnaire had been received from the participant at the earlier (4 and 8-month) time points. The concept of social incentive that underpinned the intervention for this study was that a social incentive is something that persuades people to behave in a certain way by the promise that their actions will be noticed or made public ^10^. Therefore, the cover letter was intended to highlight to the participant that their questionnaire responses are noted and valued ^10^.

**Table 1.** Intervention groups.

Pen York Trials Unit branded pen, standard cover letter *(Supplementary File 1)* *	Control Group No pen, standard cover letter *(Supplementary File 4)*.
Pen and Social Incentive Cover Letter York Trials Unit branded pen, social incentive cover letter *(Supplementary File 3)*.	Social Incentive cover letter Social incentive cover letter ( *Supplementary File 2*), no pen.

*Supplementary Files are available as Extended data ^13^.

### Blinding and quality assurance

Participants were blind to their participation. Research administrators and research team members posting the questionnaire packs were not blind to the intervention; however, administrators who recorded the outcome data were blind to allocation.

### Primary objective

To assess whether a pen and/or social incentive text cover letter sent with the 12-month questionnaire increased postal questionnaire response rates for participants in the OTIS trial.

### Primary outcome

The primary outcome was response rate, defined as the proportion of participants in each group who returned the 12-month questionnaire.

### Secondary outcomes

- Time to return 12-month questionnaire
- The completeness of the 12-month questionnaire
- The requirement for a reminder letter to be sent
- Cost effectiveness

### Statistical analysis

The data were analysed in SPSS v25 ^14^ using two-sided tests at the 5% significance level on an intention-to-treat basis. Participants who withdrew or died before the 12-month questionnaire was sent were excluded from the analysis. The primary outcome was compared using a logistic regression model adjusting for age (retention is generally higher in participants ˂75 years and older adults may respond differently to incentives ^15^), gender (to control for potential differences in anticipation of social incentives between males and females ^16^) and host trial treatment allocation. The presence of an interaction between the two interventions was tested by introducing the interaction term into the logisit model. Time to questionnaire return (calculated as days from questionnaire sent to return) was analysed using Cox Proportional Hazards regression, adjusting for the same covariates as in the primary analysis. The proportional hazards assumption was assessed using Schoenfeld residuals ^17^. Completeness of response (defined as number of items completed) was analysed by linear regression model and adjusted as for the primary analysis.

Cost effectiveness was calculated for each group using the total cost of the pen/letter/postage/stationary and staff time.

Due to SWATs typically being under-powered to show small effects, it is essential that the results are seen within the context of the wider literature. A fixed effect meta-analysis using the Mantel-Haenszel method was conducted using review manager v5.3 ^18^ to pool the results of this study for enclosing a pen with the 12-month questionnaire with other RCT evidence. These were located utilising the Cochrane systematic review ^7^ search strategy (Supplementary file 14) in MEDLINE and EMBASE, along with hand searching of previous systematic reviews references, published retention research reference lists, conference papers and co-author personal knowledge of studies. The results of this study were pooled with four previous SWATs ^8, 9, 19, 20^ investigating the same intervention, with the same dichotomous outcome of response to the questionnaire or not. Pooled odds ratios and corresponding 95% CIs were calculated. Heterogeneity between trials was assessed using the Chi-squared and I ^2^ statistics. The meta-analysis was facilitated by the PROMoting THE USE of SWATs (PROMETHEUS) programme, which supports host trial teams to conducted SWATs and for data obtained to be collated and meta-analysed.

A meta-analysis of the results of the social incentive intervention was not undertaken as the only previous study using this was conducted within a cohort study rather than an RCT ^10^.

## Results

**Figure 1.**
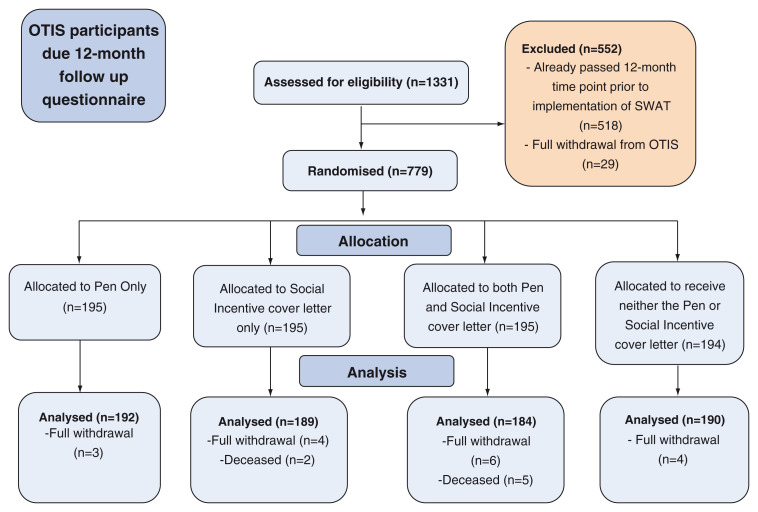
Flow diagram depicting the recruitment and retention of participants in this embedded trial.

Figure 1 depicts the recruitment and retention of participants in the embedded trial. Table 2 presents summary statistics for the baseline characteristics of the SWAT participants.

**Table 2.** Baseline characteristics of the SWAT participants.

	Pen only (n=192)	Pen and social incentive cover letter (n=184)	Social incentive cover letter only (n=189)	Standard 12- month cover letter (control) (n=190)
**Age**				
n	192	184	189	190
Mean (SD)	80 (6.3)	80 (6.1)	79 (6.2)	80 (6.2)
Min, Max	67, 98	66, 98	65, 98	69, 94
**Gender**				
n	192	184	189	190
Male	73 (38.0%)	56 (30.4%)	59 (31.2%)	69 (36.3%)
Female	119 (62.0%)	128 (69.6%)	130 (68.8)	121 (63.7%)
**Host trial randomisation**				
n	192	184	189	190
OT visit (intervention)	61 (31.8%)	49 (26.6%)	59 (31.2%)	65 (34.2%)
GP standard care	131 (68.2%)	135 (73.4%)	130 (68.8%)	125 (65.8%)
**Number of falls in 12 months prior to randomisation**				
n	145	139	149	135
Mean	2.2 (3.0)	1.8 (1.4)	2.0 (1.7)	2.2 (2.1)
Min, Max	1, 21	1, 11	1, 10	1, 15

#= How good or bad your health is today rated from 0 worst, 100 best.

### Primary outcome

Between randomisation into the SWAT and being sent their 12-month questionnaire, 24 randomised participants either died or withdrew from the host trial and so were not sent the questionnaire. A total of 721/755 (95.5%) returned the 12-month questionnaire. The response rate was identical in the pen only group (184/192, 95.8%), social incentive cover letter only group (181/189, 95.8%) and control group (182/190, 95.8%). However, it was marginally lower in the pen and social incentive cover letter group (174/184, 94.6%).

No evidence of a difference in response rates was found between participants with or without pens (pen: 358/376 [95.2%]; no pen: 363/379 [95.8%]; adjusted OR 0.90, 95% CI 0.45 to 1.80, p=0.77) nor with or without the social incentive cover letter (cover letter: 355/373 [95.2%]; no cover letter: 366/382 [95.8%]; adjusted OR 0.84, 95% CI 0.42 to 1.69, p=0.63) ( Table 3).

**Table 3.** Primary outcome results.

Primary outcome	Group	Hazard ratio (HR)/ Odds ratio (OR)/Mean difference (MD)	95% Confidence Interval	p-value	Other
**Response** **rate**	Pen received vs. not received	OR = 0.90	0.45, 1.80	0.77	Total of 721/755 (95.5%) returned tde 12-month questionnaire
	Social incentive cover letter received vs. not received	OR = 0.84	0.42, 1.69	0.29	
	Host trial allocation (intervention vs. control)	OR = 1.40	0.64, 3.23	0.38	
	Age (per year)	OR = 0.96	0.91, 1.01	0.11	
	Gender (male vs. female)	OR = 0.71	0.35, 1.44	0.35	

The interaction between the interventions was found to be non-significant (interaction effect size estimate OR 0.79 95% CI 0.20, 3.15 p = 0.74).

### Secondary outcomes

***Time to return***. Median time to return the questionnaire was nine days, with a mean of 12.2 days. No statistically significant difference between the groups was found ( Table 4).

**Table 4.** Secondary outcome results.

Secondary outcome	Group	Hazard ratio (HR)/ Odds ratio (OR)/Mean difference (MD)	95% Confidence Interval	p-value	Other
**Time to return**	Pen received vs. not received	HR = 1.08	0.93, 1.25	0.30	Mean time for all participants to return questionnaire = 12.2 days. Median time for all participants to return questionnaire = 9 days.
	Social incentive cover letter received vs. not received	HR =1.101	0.87, 1.17	0.92	
	Host trial allocation (intervention vs. control)	HR = 0.85	0.73, 1.00	0.05	
	Age (per year)	HR = 0.99	0.97, 1.00	0.02	
	Gender (male vs. female)	HR = 1.80	0.92, 1.26	0.35	
**Reminders** **sent**	Pen received vs. not received	OR = 0.89	0.56, 1.42	0.63	83/755 (11.0%) required a reminder p value associated with the Kruskal- Wallis test statistic p=0.190
	Social incentive cover letter received vs. not received	OR = 0.92	0.58, 1.47	0.74	
	Host trial allocation (intervention vs. control)	OR = 1.611	1.00, 2.59	0.05	
	Age (per year)	OR = 1.04	1.00, 1.08	0.03	
	Gender (male vs. female)	OR = 0.87	0.53, 1.42	0.57	
**Completeness** **of response**	Pen received vs. not received	MD = 0.14	-0.46, 0.74	0.65	Overall average completeness of the questionnaires was 27.8/31 questions (89.6% complete)
	Social incentive cover letter received vs. not received	MD = 0.09	-0.69, 0.51	0.78	
	Host trial allocation (intervention vs. control)	MD = -0.10	-0.55, 0.75	0.77	
	Age (per year)	MD = -0.10	-0.46, 0.74	0.65	
	Gender (male vs. female)	MD = -1.06	-1.69, -0.42	˂0.001	

***Reminders sent***. In total, 83/755 (11.0%) participants required a reminder letter. The pen and social incentive cover letter group required the least reminders (19/184 10.3%) and the control group required the most reminders (24/190 12.6%). No statistically significant evidence was found of a difference of participants requiring a reminder between the groups ( Table 4).

***Completeness of response***. Overall average completeness of the questionnaires was 27.8/31 questions (89.6% complete) with no evidence of a difference in completeness of the questionnaire between pen received or not ( Table 4).

***Cost effectiveness***. Due to the non-statistically significant effect of the interventions on response rates calculating overall associated costs provides evidence of potential cost savings not to send the social incentive cover letter and/or pen ( *Extended data:* Supplementary File 9 ^13^).

### Meta-analysis

**Figure 2.**
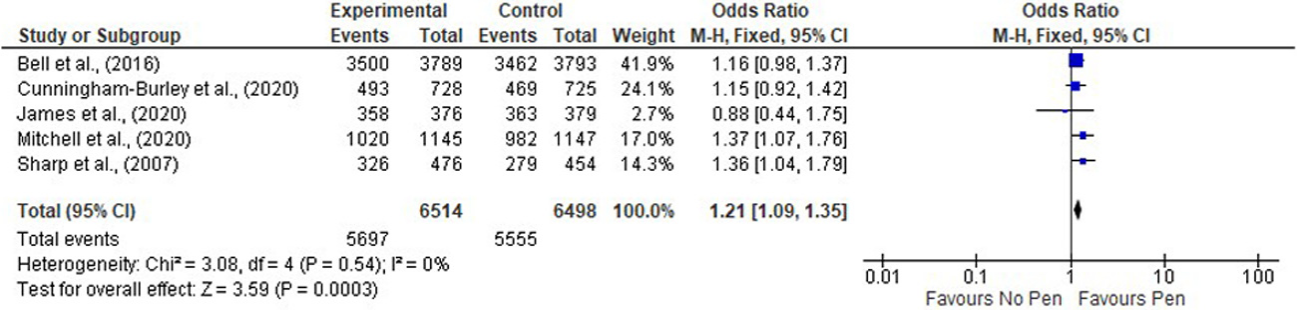
Meta-analysis of enclosing a pen with a questionnaire to increase response rate to a postal questionnaire.

A fixed effect meta-analysis of enclosing a pen with a follow-up postal questionnaire on response rate was conducted ( Figure 2). This included five studies ^8, 9, 21, 22^ (n=13012 participants) and gave a statistically significant pooled OR favouring the intervention (1.21, 95% CI 1.09 to 1.35 p = 0.0004). Negligible heterogeneity was observed (chi-squared = 2.88 I ^2^= 0%). The risk of bias was low, as indicated by the Cochrane’s risk of bias tool assessment undertaken ^23^ ( *Extended data:* Supplementary File 10 ^13^).

## Discussion

This SWAT found no evidence that sending a pen and/or a social incentive cover letter with a postal, trial follow-up questionnaire improved response rate, time to return, requirement for a reminder, or questionnaire completeness.

A limitation was the average age of the participants (79.9 years) giving a narrow age demographic thus restricting generalisability of results. Further investigation of the pen and social incentive cover letter in RCTs are required across more diverse populations.

The OTIS trial hosted three other methodological SWATs; therefore, there was a potential for contamination or interaction. It is preferable to plan all SWATs that will be undertaken in the early design stages ^19^, to ensure they are planned accordingly to reduce the potential of this.

The overall response rate of the 12-month postal questionnaire for all SWAT participants was 95.7%, which may have been helped by the inclusion of £5 to all participants as standard. This high response rate is therefore difficult to improve upon, furthermore the incentives may not have been as effective with participants who are very committed to the behaviour ^10^. The incentive required for committed participants may be different ^10, 20^. A learning point being that future SWATS testing these interventions should avoid doing so in trials with already high response rates.

## Conclusion

Whilst neither the pen nor the social incentive cover letter showed an effect on response rate, the meta-analysis evidence remains that including a pen increases response rates. This reinforces that for interventions where small effects are likely, it is important to undertake a number of trials and combine these to be confident of an intervention’s effectiveness. Further investigation of the social incentive cover letter in RCTs is required to determine effectiveness.

## Data availability

### Underlying data

Open Science Framework: Pen and Social Incentive Cover Letter Retention SWAT, https://doi.org/10.17605/OSF.IO/7TDRB ^13^.

### Extended data

Open Science Framework: Pen and Social Incentive Cover Letter Retention SWAT, https://doi.org/10.17605/OSF.IO/7TDRB ^13^.

This project contains the following extended data:

- Full study protocol
- Supplementary File 1- Cover letter for the Pen only group.
- Supplementary File 2 - Cover letter for the Social incentive cover letter only group.
- Supplementary File 3 - Cover letter for the Pen and social incentive cover letter group.
- Supplementary File 4 - Cover letter for the control group.
- Supplementary File 5 - Results table by intervention group
- Supplementary File 6 - Graph Survival curve of pen vs no pen and time taken to return 12-month questionnaire.
- Supplementary File 7 - Graph Survival curve of Social incentive cover letter vs no social incentive cover letter and time taken to return 12-month questionnaire.
- Supplementary File 8- Survival curve of host trial allocation and time taken to return 12-month questionnaire.
- Supplementary File 9 – Costings table
- Supplementary File 10 – Cochrane Risk of bias tool assessments for Bell *et al.,* (2016) ^8^, Sharp *et al.,* (2006) ^9^, Cunningham-Burley *et al.,* (2020) ^21^, Mitchell *et al.,* (2020) ^22^ and James *et al.,* (2020).
- Supplementary File 11 – Summary of all SWATs undertaken in the OTIS study
- Supplementary File 12 – Copy of the OTIS reminder letter
- Supplementary file 13 – Summary of studies included in the meta-analysis
- Supplementary File 14 – Copy of the search strategies of Brueton *et al*., (2014)

### Reporting guidelines

Open Science Framework: CONSORT checklist for ‘Including a pen and/or cover letter, containing social incentive text, had no effect on questionnaire response rate: a factorial randomised controlled Study within a Trial’,  ^13^.

Data are available under the terms of the Creative Commons Zero "No rights reserved" data waiver (CC0 1.0 Public domain dedication).
