# Supplementary material for: Including a pen and/or cover letter, containing social incentive text, had no effect on questionnaire response rate: a factorial randomised controlled Study within a Trial
Source: F1000Res. 2021 Nov 8;9:623. Originally published 2020 Jun 17. [Version 2] doi: 10.12688/f1000research.23767.2 (PMC8772525; doi:10.12688/f1000research.23767.2)
Supplement: Peer review history

# Peer review: Reviewer response for version 2

**DOI:** 10.5256/f1000research.77829.r99236

**Published:** 2022-01-19

**Authors:** Eilidh Duncan, Louisa Lawrie, Taylor Coffey

**Affiliations:** 1 Health Services Research Unit, University of Aberdeen, Aberdeen, UK

Comments have been addressed.

Is the work clearly and accurately presented and does it cite the current literature?

Yes

If applicable, is the statistical analysis and its interpretation appropriate?

Yes

Are all the source data underlying the results available to ensure full reproducibility?

Yes

Is the study design appropriate and is the work technically sound?

Yes

Are the conclusions drawn adequately supported by the results?

Yes

Are sufficient details of methods and analysis provided to allow replication by others?

Yes

Reviewer Expertise:

Health services research, design and evaluation of interventions to change behaviour; the application of behavioural theory to trial recruitment and retention.

We confirm that we have read this submission and believe that we have an appropriate level of expertise to confirm that it is of an acceptable scientific standard.

# Peer review: Reviewer response for version 2

**DOI:** 10.5256/f1000research.77829.r99235

**Published:** 2021-12-06

**Authors:** Susanne Grylka-Baeschlin

**Affiliations:** 1 Research Unit for Midwifery Science, Zurich University of Applied Sciences, Katharina-Sulzer-Platz, Winterthur, Switzerland

Thank you for thoroughly revising this manuscript. The limitations of the study are now addressed in the discussion, nevertheless diminish the importance of the results. However, the significance of the study was increased by the meta-analysis. I only have one very minor comment:

- Table 3, column “Other”: should it be “the…” instead of “tde 12-month questionnaire…”?

Is the work clearly and accurately presented and does it cite the current literature?

Yes

If applicable, is the statistical analysis and its interpretation appropriate?

Yes

Are all the source data underlying the results available to ensure full reproducibility?

Yes

Is the study design appropriate and is the work technically sound?

Partly

Are the conclusions drawn adequately supported by the results?

Partly

Are sufficient details of methods and analysis provided to allow replication by others?

Yes

Reviewer Expertise:

Midwifery, Epidemiology

I confirm that I have read this submission and believe that I have an appropriate level of expertise to confirm that it is of an acceptable scientific standard.

# Peer review: Reviewer response for version 1

**DOI:** 10.5256/f1000research.26227.r91091

**Published:** 2021-08-16

**Authors:** Susanne Grylka-Baeschlin

**Affiliations:** 1 Research Unit for Midwifery Science, Zurich University of Applied Sciences, Katharina-Sulzer-Platz, Winterthur, Switzerland

Thank you very much for giving me the opportunity to review this article. Studies Within A Trial (SWAT) are important and a good opportunity to make optimal use of resources. Despite their disadvantage that the original trial has not been designed for the purpose of the SWAT, they provide important indications for further studies. In this sense, the results of this SWAT are important for further research.

**General impression:**

 This SWAT was planned at the same time as the original trial. The methods are described in detail and are the strongest part of the article. Nevertheless, there are some important concerns. Participants were rewarded with a 5-pound note. This is in itself also an intervention to increase response rates. As the authors themselves note, the response rate is generally very high. Possibly the additional reward and together with the high response rate made it impossible to show an effect of the two tested interventions.

**Abstract:**

- Background: Is it still true that postal questionnaires are used frequently in randomized controlled trials? One could imagine that a swift towards online surveys can be observed.

**Background:**

- This very short background is the weakest part of the manuscript.
- The rational for the SWAT differs between the abstract and the main text. The general problem of low response rate for the generalizability of results seems important also for the article, not only for the abstract.

**Methods:**

- This is the strongest part of the article and mostly very clear.
- It was a good idea to combine the results of this SWAT with results of other studies in a meta-analysis. However, it could be meaningful to provide further details about the literature research for the articles, which were included. There is not enough information to decide if the research was really systematic or not. Especially the comment that co-author personal knowledge of studies was considered for finding studies raises some questions. Did the included articles test the same interventions?

**Results:**

- Results are presented in a clear way with nice tables. Especially Figure 1 which gives very good overviews about the study process.
- Are differences between the study groups described in Table 2 significant or not?

**Discussion:**

- The enclosed 5-pound note represents a further limitation and should be mentioned.

Is the work clearly and accurately presented and does it cite the current literature?

Yes

If applicable, is the statistical analysis and its interpretation appropriate?

Yes

Are all the source data underlying the results available to ensure full reproducibility?

Yes

Is the study design appropriate and is the work technically sound?

Partly

Are the conclusions drawn adequately supported by the results?

Partly

Are sufficient details of methods and analysis provided to allow replication by others?

Yes

Reviewer Expertise:

Midwifery, Epidemiology

I confirm that I have read this submission and believe that I have an appropriate level of expertise to confirm that it is of an acceptable scientific standard, however I have significant reservations, as outlined above.

# Response

**Published:** 2021-09-20

**Authors:** Sophie James

**Affiliations:** University of York, UK, UK

We thank the reviewer for the helpful comments and points raised. We hope that our responses are satisfactory and the article has been updated in response to the points raised from both reviewers (v2).

**General impression:**

**This SWAT was planned at the same time as the original trial. The methods are described in detail and are the strongest part of the article. Nevertheless, there are some important concerns. Participants were rewarded with a 5-pound note. This is in itself also an intervention to increase response rates. As the authors themselves note, the response rate is generally very high. Possibly the additional reward and together with the high response rate made it impossible to show an effect of the two tested interventions.**

*Response: Thank you, these are valid points. Brueton et al., (2013) report that the addition of a monetary incentive was more effective than no incentive (RR 1.31; 95% CI 1.11 to 1.55, p=0.002) so the £5 monetary reward was included as standard in the OTIS trial at the 12 month time point.  All participants received the £5 so this was not felt to be a limitation in assessing the effectiveness of the 2 interventions investigated by this SWAT, but it may have added to the high response rate and this is now noted in the discussion. At the time this SWAT was designed and added to the OTIS trial the high response rates were not known. The high response rate is mentioned in the discussion of the paper with a learning point for future SWATs, as we acknowledge that this limits the scope for the interventions to have a large benefit.*

**Abstract:**

- **Background: Is it still true that postal questionnaires are used frequently in randomized controlled trials? One could imagine that a swift towards online surveys can be observed.**

*Response: We believe it is still true, indeed most of the trials we conduct in York Trial Unit still use postal questionnaires; however, we agree that the use of online data collection has increased since this SWAT was designed 4 years ago. With RCTs in this demographic/population and for participants in RCTs who do not have internet provision, in order to ensure inclusivity, there is likely to remain a need for future RCTs to still have a postal paper questionnaire option available and so keeps this relevant.*

**Background:**

- **This very short background is the weakest part of the manuscript.**
- **The rational for the SWAT differs between the abstract and the main text. The general problem of low response rate for the generalizability of results seems important also for the article, not only for the abstract.**

*Response: Thank you for this, we have updated the paper v2 to include this information from the abstract into the introduction to ensure consistency. We were limited by the word count of the article to be able to provide any further detail in this section.*

**Methods:**

- **This is the strongest part of the article and mostly very clear.**
- **It was a good idea to combine the results of this SWAT with results of other studies in a meta-analysis. However, it could be meaningful to provide further details about the literature research for the articles, which were included. There is not enough information to decide if the research was really systematic or not. Especially the comment that co-author personal knowledge of studies was considered for finding studies raises some questions. Did the included articles test the same interventions?**

*Response: All the studies included in the meta-analysis are now published and peer reviewed with the references provided so that these can be located and a summary of the key information of the studies included in the meta analysis is now provided in Supplementary file 13.*

*[Added to paper v2: The Cochrane systematic review search strategy (supplementary file 14) was used to search MEDLINE and EMBASE. Additionally previous systematic reviews references were hand searched, along with published retention research reference lists, conference papers and co-author personal knowledge of studies.]*

**Results:**

- **Results are presented in a clear way with nice tables. Especially Figure 1 which gives very good overviews about the study process.**
- **Are differences between the study groups described in Table 2 significant or not?**

*Response: Table 2 presents summary statistics for the baseline characteristics of the SWAT participants. According to the CONSORT statement, significance testing of baseline differences in randomized controlled trials should not be performed. Indeed, this practice has been discouraged by numerous authors.*

*(Reference for the above information =* *Michiel R de Boer* *,* *Wilma E Waterlander* *,* *Lothar DJ Kuijper* *,* *Ingrid HM Steenhuis* *, and* *Jos WR Twisk* *(2015) Testing for baseline differences in randomized controlled trials: an unhealthy research behavior that is hard to eradicate. Int J Behav Nutr Phys Act. 2015; 12: 4.Published online 2015 Jan 24. doi:* *10.1186/s12966-015-0162-z* *)*

**Discussion:**

- **The enclosed 5-pound note represents a further limitation and should be mentioned.**

*Now mentioned in the discussion*

# Peer review: Reviewer response for version 1

**DOI:** 10.5256/f1000research.26227.r66639

**Published:** 2020-07-21

**Authors:** Eilidh Duncan, Taylor Coffey, Louisa Lawrie

**Affiliations:** 1 Health Services Research Unit, University of Aberdeen, Aberdeen, UK

The present article by James *et al.* details a study within a trial (SWAT) of including a pen and/or cover letter with a postal questionnaire. A two-by-two factorial randomized control trial was conducted that assessed the effect of including a branded pen, a ‘social incentive’ cover letter, both a pen and ‘social incentive’ cover letter, or a standard cover letter as control. The primary outcome measure was the return of a postal questionnaire with secondary outcomes of time to return the questionnaire, completeness of the questionnaire, requirement for a reminder letter to be sent, and overall cost effectiveness. Overall, there was no effect of any intervention on return of questionnaires and no effect on secondary outcome measures. A meta-analysis was conducted with respect to including a pen with the questionnaire and its effect on response rate, with pooled results supporting the inclusion of pens.

 We would firstly like to commend the authors for presenting the methods clearly and providing supplementary material which enhances replicability. Below we indicate points which could be addressed and identify areas of improvement to further enhance the quality of this article.

*Major points to address: The peer review team would like to highlight the following areas for clarification.*

 Potentially confounding incentives

- The ‘social incentive’ intervention cover letter is described as including a “mild level of social incentive”. In referencing the supporting citation for the cover letter (Cotterill *et al*., 2017) ^1^, the social incentive used is social pressure. Social pressure is described as conveying to participants that “information about their behavior will be noticed (Cotterill *et al*., 2017) ^1^”. The present paper then describes the cover letter as “intended to highlight to the participant that their questionnaire responses are noted and valued”. It is apparent that reminder letters were also sent to some participants. A reminder letter could be argued to be another “mild level of social incentive” as it is similarly conveying to participants that their behaviour (i.e. not returning the questionnaire) is being noticed. Can the authors comment on the potential for the reminder letters to confound the results? The reminder is not included among the supplementary documents, but we would be interested in the authors’ thoughts on the potential for unintended social pressure cues or behaviour change techniques being delivered through the reminder letter.
- In reviewing both the standard and social incentive cover letter, it is noted that they are identical apart from the personalized table indicating the participants’ previous responses. Similarly to the above point regarding unintended social pressure cues, we wonder if the phrases “We would be very grateful if you could return the completed questionnaire to us[…]” and “Your input to this trial is very important to us[…]”can be unintentionally introducing confounding social pressure by highlighting that their questionnaire responses are “noted and valued”. Can the authors reflect on how the addition of these phrases (and thereby the possibility that unintended ‘social incentives’ were applied within the control group) may have potentially affected results?
- Under the section, “Interventions”, on page 3, it is mentioned that an unconditional £5 note was included with the questionnaire for all participants. Due to the financial incentive present, we would just like to clarify if the inclusion of the £5 note was controlled for in the analysis.
- It is referenced in the “Discussion” section that the host trial included three other methodological SWATs that have a potential for contamination or interaction. If possible, it would be good to offer brief descriptions or links to other SWATS in the supplementary materials to better understand their potential to confound intervention effects.

Theoretical foundation of cover letter

- Applying the Behaviour Change Techniques (BCT) taxonomy definitions to the intervention letter raises the possibility that the intervention being tested is more akin to “feedback on behaviour” (see Michie, 2013) ^2^, given that the letter provides informative feedback on the frequency of questionnaire return, rather than the BCT taxonomy definition of ‘social incentive’ (i.e. “Inform that a verbal or nonverbal reward will be delivered if and only if there has been effort and/or progress in performing the behaviour"). It is worth noting that some readers may interpret the meaning of 'social incentives' quite differently than how it is conceptualised here. Can the authors offer some clarification on this for the reader and on the theoretical underpinnings of the intervention? Adding citation(s) to clarify their conceptualisation of 'social incentive' would be helpful.
- It is also unclear whether the authors regard the social incentive cover letter as a form of “social reward”: noted on page 4, the authors state that gender was controlled for within the logistic regression model to “control for potential differences in anticipation of social rewards…”. It does not appear that participants are presented with additional verbal or non-verbal reward above the standard letter with just the inclusion of the response table.
- It is noted in Cotterill *et al*. (2017) ^1^ that social pressure is “unlikely to be effective among those who are very committed to the behavior” which appears to be the case given that response rates across groups are >95%. Given that there are two prior postal response timepoints, was identifying if this group is highly committed to questionnaire response considered?

Minor points to address:

- Regarding Table 2, are BMI, EQ-55D-5L score, and number of falls relevant to include? The table is already quite extensive and this data may detract the reader’s attention from other variables (e.g. age, sex) more pertinent to the study.
- The pen is described as York Trials Unit branded. Was there branding specific to the host trial? It might be that participants would be quicker to recognize the trial versus the CTU. How might this affect the social incentive?
- Could it be mentioned briefly in the main body of the paper that the meta-analysis was undertaken as part of PROMETHEUS? We notice it is mentioned in the acknowledgments but one of the review team members was confused about the inclusion of the meta-analysis.

*Overall assessment: We would like to commend the authors on a concise and well-articulated presentation of the present study. The study is well designed and it is clearly presented with accompanying data and figures making it highly reproducible. We recognize the importance of each individual SWAT’s contribution to a larger body of evidence and the conclusions drawn and the recommendations for future work are appropriate. We have suggested potential limitations and concerns with the theoretical foundations of the social incentive cover letter and the presence of potential confounding incentives but our overall assessment is that the article meets its objectives and its strengths merit indexing.*

*Contribution to this peer review:*

 This peer review was conducted by Taylor Coffey, Dr Louisa Lawrie and Dr Eilidh Duncan as part of TC and LL’s training. All reviewers contributed to writing the report.

Is the work clearly and accurately presented and does it cite the current literature?

Yes

If applicable, is the statistical analysis and its interpretation appropriate?

Yes

Are all the source data underlying the results available to ensure full reproducibility?

Yes

Is the study design appropriate and is the work technically sound?

Yes

Are the conclusions drawn adequately supported by the results?

Yes

Are sufficient details of methods and analysis provided to allow replication by others?

Yes

Reviewer Expertise:

Health services research, design and evaluation of interventions to change behaviour; the application of behavioural theory to trial recruitment and retention.

We confirm that we have read this submission and believe that we have an appropriate level of expertise to confirm that it is of an acceptable scientific standard, however we have significant reservations, as outlined above.

## References

[1] : The effect of using social pressure in cover letters to improve retention in a longitudinal health study: an embedded randomised controlled retention trial. *Trials* .2017;18(1) : 10.1186/s13063-017-2090-5 10.1186/s13063-017-2090-5 28728603PMC5520383

[2] : The behavior change technique taxonomy (v1) of 93 hierarchically clustered techniques: building an international consensus for the reporting of behavior change interventions. *Ann Behav Med* .2013;46(1) : 10.1007/s12160-013-9486-6 81-95 10.1007/s12160-013-9486-6 23512568

# Response

**Published:** 2021-09-20

**Authors:** Sophie James

**Affiliations:** University of York, UK, UK

***Major points to address:*** ***The peer review team would like to highlight the following areas for clarification.***

**Potentially confounding incentives**

- **The ‘social incentive’ intervention cover letter is described as including a “mild level of social incentive”. In referencing the supporting citation for the cover letter (Cotterill *et al*., 2017)** **1** **, the social incentive used is social pressure. Social pressure is described as conveying to participants that “information about their behavior will be noticed (Cotterill *et al*., 2017)** **1** **”. The present paper then describes the cover letter as “intended to highlight to the participant that their questionnaire responses are noted and valued”. It is apparent that reminder letters were also sent to some participants. A reminder letter could be argued to be another “mild level of social incentive” as it is similarly conveying to participants that their behaviour (i.e. not returning the questionnaire) is being noticed. Can the authors comment on the potential for the reminder letters to confound the results? The reminder is not included among the supplementary documents, but we would be interested in the authors’ thoughts on the potential for unintended social pressure cues or behaviour change techniques being delivered through the reminder letter.**                             

*Thank you to the reviewers for their valued comments.*

*For this paper the term ‘social incentive’ was used rather than ‘social pressure’ on the advice of two of the co-authors of this paper who were involved in the original Cotterill et al., (2017) embedded trial to test the social incentive cover letter intervention within a cohort study. They noted that following this paper the term ‘social pressure’ is not commonly used outside of politics literature.*

*A copy of the reminder letter that was sent to 83 participants has been added to the OSF as supplementary document 12.*

*Whilst it could be argued that the reminder letter provided another mild level of social incentive, it was not felt that it would provide additional social incentive as per the definition used for this study (discussed in relation to the next comment).  Furthermore, the number of these sent out was evenly distributed across the randomised and analysed groups: 83 reminder letters sent were sent out of 755 and these were evenly distributed across the 4 groups (pen and social incentive cover letter group 19/184 (10.3%), control group 24/190 (12.6%), social incentive cover letter only group 20/189 (10.5%) and pen only group 20/192 (10.4%). So it would be unlikely that these additional reminder letters would confound the results.*

*There was no statistically significant evidence of a difference in the likelihood of participants requiring a reminder between the groups (pen vs no pen: adjusted OR = 0.89 [95% CI 0.56, 1.42 p = 0.63]; social incentive cover letter vs no social incentive cover letter: adjusted OR = 0.92 [95% CI 0.58, 1.47 p = 0.74].*

- **In reviewing both the standard and social incentive cover letter, it is noted that they are identical apart from the personalized table indicating the participants’ previous responses. Similarly to the above point regarding unintended social pressure cues, we wonder if the phrases “We would be very grateful if you could return the completed questionnaire to us[…]” and “Your input to this trial is very important to us[…]”can be unintentionally introducing confounding social pressure by highlighting that their questionnaire responses are “noted and valued”. Can the authors reflect on how the addition of these phrases (and thereby the possibility that unintended ‘social incentives’ were applied within the control group) may have potentially affected results?**

*The standard text for this cover letter was the exact text used in the cover letters previously sent to participants with their 4 and 8 month questionnaires for the OTIS trial. The text of both the ‘control’ and ‘social incentive’ cover letters were identical the only difference was the addition of the table therefore it was not felt that this would affect the results. The example sentences noted could fall into some definitions for social incentive, but they would not meet ‘persuades people to behave in a certain way by the promise that their actions will be noticed or made public’ (Cotterill et al., 2017). Clarification of this definition has been added to the paper to help to clarify the comments around this.*

  

- **Under the section, “Interventions”, on page 3, it is mentioned that an unconditional £5 note was included with the questionnaire for all participants. Due to the financial incentive present, we would just like to clarify if the inclusion of the £5 note was controlled for in the analysis.**

*Since all participants received the £5 it was neither necessary nor appropriate to control for this in the analysis.*

  

- **It is referenced in the “Discussion” section that the host trial included three other methodological SWATs that have a potential for contamination or interaction. If possible, it would be good to offer brief descriptions or links to other SWATS in the supplementary materials to better understand their potential to confound intervention effects.**

*A supplementary file 11 has been added to OSF outlining the other SWATs that were undertaken within the OTIS Trial.*

**Theoretical foundation of cover letter**

- **Applying the Behaviour Change Techniques (BCT) taxonomy definitions to the intervention letter raises the possibility that the intervention being tested is more akin to “feedback on behaviour” (see Michie, 2013)** **2** **, given that the letter provides informative feedback on the frequency of questionnaire return, rather than the BCT taxonomy definition of ‘social incentive’ (i.e. “Inform that a verbal or nonverbal reward will be delivered if and only if there has been effort and/or progress in performing the behaviour"). It is worth noting that some readers may interpret the meaning of 'social incentives' quite differently than how it is conceptualised here. Can the authors offer some clarification on this for the reader and on the theoretical underpinnings of the intervention? Adding citation(s) to clarify their conceptualisation of 'social incentive' would be helpful.**

*Although recent work has pointed towards the importance of using behaviour change theory to support and influence behavioural actions required by participants in RCTs (Gilles et al., 2018), behaviour change theory and associated research is such a vast area that this is a valid point that different concepts can be defined in different ways by different theories/authors. To help to clarify this in the paper sentences have been added to provide further detail and detail how the concept of social incentive was interpreted in relation to this study.*

*[added to paper v2: ‘The concept of social incentive that underpinned the intervention for this study was that a social incentive is something that persuades people to behave in a certain way by the promise that their actions will be noticed or made public [10].’*

- **It is also unclear whether the authors regard the social incentive cover letter as a form of “social reward”: noted on page 4, the authors state that gender was controlled for within the logistic regression model to “control for potential differences in anticipation of social rewards…”. It does not appear that participants are presented with additional verbal or non-verbal reward above the standard letter with just the inclusion of the response table.**

*Error in terminology of the paper, social reward was used interchangeably with social incentive in error. Social reward removed from this sentence and replaced with social incentive with a further reference added.*

*[changed paper v2 to read: ‘gender, to control for potential difference in the effect of social incentives between males and females]*

  

- **It is noted in Cotterill *et al*. (2017)** **1** **that social pressure is “unlikely to be effective among those who are very committed to the behavior” which appears to be the case given that response rates across groups are >95%. Given that there are two prior postal response timepoints, was identifying if this group is highly committed to questionnaire response considered?**

*Thank you, this is a valid point.  At the time this SWAT was designed and added to the OTIS trial the high response rates of the previous time points were not known. This is currently noted in the paper as a consideration for future research to consider when using this type of social incentive cover letter.*

**Minor points to address:**

- **Regarding Table 2, are BMI, EQ-55D-5L score, and number of falls relevant to include? The table is already quite extensive and this data may detract the reader’s attention from other variables (e.g. age, sex) more pertinent to the study.**

*These variables have been removed from Table 2 as suggested.*

  

- **The pen is described as York Trials Unit branded. Was there branding specific to the host trial? It might be that participants would be quicker to recognize the trial versus the CTU. How might this affect the social incentive?**

*The pen had the logo of York Trials Unit and was not specific to the OTIS trial. This would be an interesting follow on question for future work, whether the branding of the pen sent with a questionnaire had any effect on response rate. The pen was not considered to provide a social incentive for this SWAT, only the cover letter.*

  

- **Could it be mentioned briefly in the main body of the paper that the meta-analysis was undertaken as part of PROMETHEUS? We notice it is mentioned in the acknowledgments but one of the review team members was confused about the inclusion of the meta-analysis.**

*[Added to paper v2: ‘Due to SWATs typically being under-powered to show small effects, it is essential that the results are seen within the context of the wider literature (added to methods section under statistical analysis)*

*The results of this study were pooled with four previous SWATs [8,9,22,23] investigating the same intervention, with the same dichotomous outcome of response to the questionnaire or not. Pooled odds ratios and corresponding 95% CIs were calculated. Heterogeneity between trials was assessed using the Chi-squared and I 2 statistics. The meta-analysis was facilitated by the PROMoting THE USE of SWATs (PROMETHEUS) programme, which supports host trial teams to conducted SWATs and for data obtained to be collated and meta-analysed.’(Summary details for the studies included in the meta-analysis are provided in Supplementary file 13)]*
